# Human Factor Considerations in Using Personal Protective Equipment in the COVID-19 Pandemic Context: Binational Survey Study

**DOI:** 10.2196/19947

**Published:** 2020-06-17

**Authors:** Avi Parush, Oren Wacht, Ricardo Gomes, Amit Frenkel

**Affiliations:** 1 Israel Institute of Technology Haifa Israel; 2 Faculty of Health Sciences Ben-Gurion University of the Negev Beer Sheva Israel; 3 Department of Operational Medicine Clinical Center of Guarda Nacional Republicana Lisboa Portugal; 4 Intensive Care Unit Soroka University Medical Center Beer Sheva Israel

**Keywords:** COVID-19, personal protective equipment, PPE, human factors, cognitive functioning, multinational survey, pandemic, protection, infectious disease, infection, survey

## Abstract

**Background:**

Full level 1 personal protective equipment (PPE) is used in various domains and contexts. Prior research has shown influences of such equipment on performance, comfort, and contamination levels. The coronavirus disease (COVID-19) pandemic forced a pervasive requirement of PPE, with little preparation, rushed deployment, inadequate time for training, and massive use by personnel who are inexperienced or not qualified in its effective use.

**Objective:**

This study aims to examine the key human factors (physical and ergonomic, perceptual and cognitive) that influence the use of level 1 PPE when attending to patients with suspected or confirmed COVID-19.

**Methods:**

The research approach consisted of a short survey disseminated to health care professionals in two countries, Israel and Portugal, with similar demographics and health care systems. The survey included 10 items with a 5-point Likert scale regarding the key human factors involved in level 1 PPE, as identified in prior research.

**Results:**

A total of 722 respondents from Israel and 301 respondents from Portugal were included in the analysis. All the respondents reported using level 1 PPE with patients with COVID-19 in the range of several hours daily to several hours weekly. The Cronbach α was .73 for Israel and .75 for Portugal. Responses showed high levels of difficulty, with medians of 4 for items related to discomfort (n=539/688, 78% in Israel; n=328/377, 87% in Portugal), hearing (n=236/370, 64% in Portugal; n=321/642, 50% in Israel), seeing (n=697/763, 89% in Israel; n=317/376, 84% in Portugal), and doffing (n=290/374, 77% in Portugal; n=315/713, 44% in Israel). A factor analysis showed a set of strongly related variables consisting of hearing, understanding speech, and understanding the situation. This suggests that degradation in communication was strongly associated with degradation in situational awareness. A subsequent mediation analysis showed a direct effect of PPE discomfort on situational awareness (*P*<.001); this was also influenced (mediated) by difficulties in communicating, namely in hearing and understanding speech.

**Conclusions:**

In 2020, the COVID-19 pandemic is paving the way for updating PPE design. The use of already deployed technology affords ample opportunities to improve, adapt, and overcome caveats. The findings here suggest that the use of level 1 PPE with patients with COVID-19 has perceptual and cognitive effects, in addition to physical and ergonomic influences. Efforts should be taken to mitigate the harmful effects of such influences, both regarding the performance of medical actions and the risk of contamination to health care workers. Such efforts involve the design of PPE; the introduction of technologies to enhance vision, hearing, and communicating during the use of PPE; and training staff in using the equipment and in effective communication and teamwork protocols.

## Introduction

Starting in December 2019 and during the first months of the year 2020, the global outbreak of the coronavirus disease (COVID-19) has forced health care professionals of various disciplines in hospital and community settings to use full level 1 personal protective equipment (PPE) to avoid contamination from patients with suspected or confirmed COVID-19 [1]. Such PPE typically consists of a completely encapsulated suit and a self-contained breathing apparatus, such as the N95 face mask, which can provide full skin, eye, and respiratory protection.

The pervasive requirement to use PPE due to the COVID-19 pandemic emerged with little preparation and rushed deployment, inadequate time for training, and massive use by personnel who are inexperienced or not qualified in the effective use of PPE. Such unique and urgent circumstances call for an examination of the use of the full PPE. Moreover, the current widespread use of PPE is not limited to contact with patients with COVID-19 in dedicated and isolated units, but rather in a variety of contexts, and in the general community.

Previous research on using full PPE in various contexts (chemical, biological, radiological, nuclear and explosive incidents, firefighting, health care, and the military) has shown several human factor problems. Much of the research addressed procedural problems, including failures to effectively put on (don) or remove (dof) the PPE [2-5], and ergonomic problems such as poor fit and discomfort while having it on [6-9]. Problems of ineffective use or decreased adherence to using the PPE were found to be associated with insufficient training and lack of prior experience [10-13], and with the appropriateness of organizational culture [12,14-16].

The use of PPE can also influence perceptual and cognitive functioning, although less research has been conducted about such influences. PPE can degrade visual perception [17]; auditory perception [18,19]; gait and balance, which are related to vision and hearing [20,21]; and cognitive functioning [22,23], communication, and teamwork [8,17,24]. Finally, according to some research, the use of PPE can result in ineffective protection against contamination [25,26]. Moreover, even effective use of PPE can influence medical actions including lifesaving procedures [13,27-29].

Taken together the use of PPE, whether effective or ineffective, has been shown to influence the user’s functioning and performance, as well as protection from contamination. Yet, the COVID-19 pandemic has introduced unique circumstances and challenges regarding the use of PPE, which prompt a critical need to re-examine the influences. This study explored these influences by means of a binational survey. In particular, we set out to identify relations of physical and ergonomic factors with perceptual and cognitive factors in the use of PPE during the COVID-19 pandemic. For this study, we considered two small developed countries that used equivalent PPE levels to deal with the COVID-19 pandemic: Israel and Portugal. The countries are similar in population, median age, and life expectancy, and have well-ranked national health care systems (NHS), 25th and 31st, respectively, in a 2018 report by the UK Health Foundation [30].

## Methods

### The Two Countries

Israel has an area of 22,145 km^2^, a population of 9,190,000, and a current density of 400 inhabitants per km^2^. The median age is 30.5 years and the life expectancy is 83.5 years. The Israeli Ministry of Health is responsible for managing the NHS by means of a public health system. The national emergency medical service is called Magen David Adom. Activated by the emergency number 101 under the coordination of the dispatch centers, specialized resources operate after triage of prehospital levels of care: basic or advanced life-support motorcycles (1 emergency medical technician [EMT] or 1 paramedic), basic life support ambulances (1 or 2 EMTs), intensive care ambulances or helicopters (1 or 2 paramedics and 1 EMT in an ambulance) [31].

Portugal has an area of 92,212 km^2^, a population of 10,202,166, and a current density of 111 inhabitants per km^2^. The median age is 46.2 years and the life expectancy is 81.5 years. The Portuguese Ministry of Health is responsible for managing the NHS, tendential and free for all residents. The main emergency medical service is managed by the National Medical Emergency Institute. Activated by the emergency number 112 under the coordination of the dispatch centers, specialized resources operate after triage of prehospital level of care: basic life support motorcycles (1 EMT), basic life support ambulances (2 EMTs), intermediate life support ambulances (1 EMT and 1 nurse), advanced critical care fast cars (1 physician and 1 nurse), and helicopter ambulances (1 physician and 1 nurse plus 2 pilots) [32].

**Figure 1:**
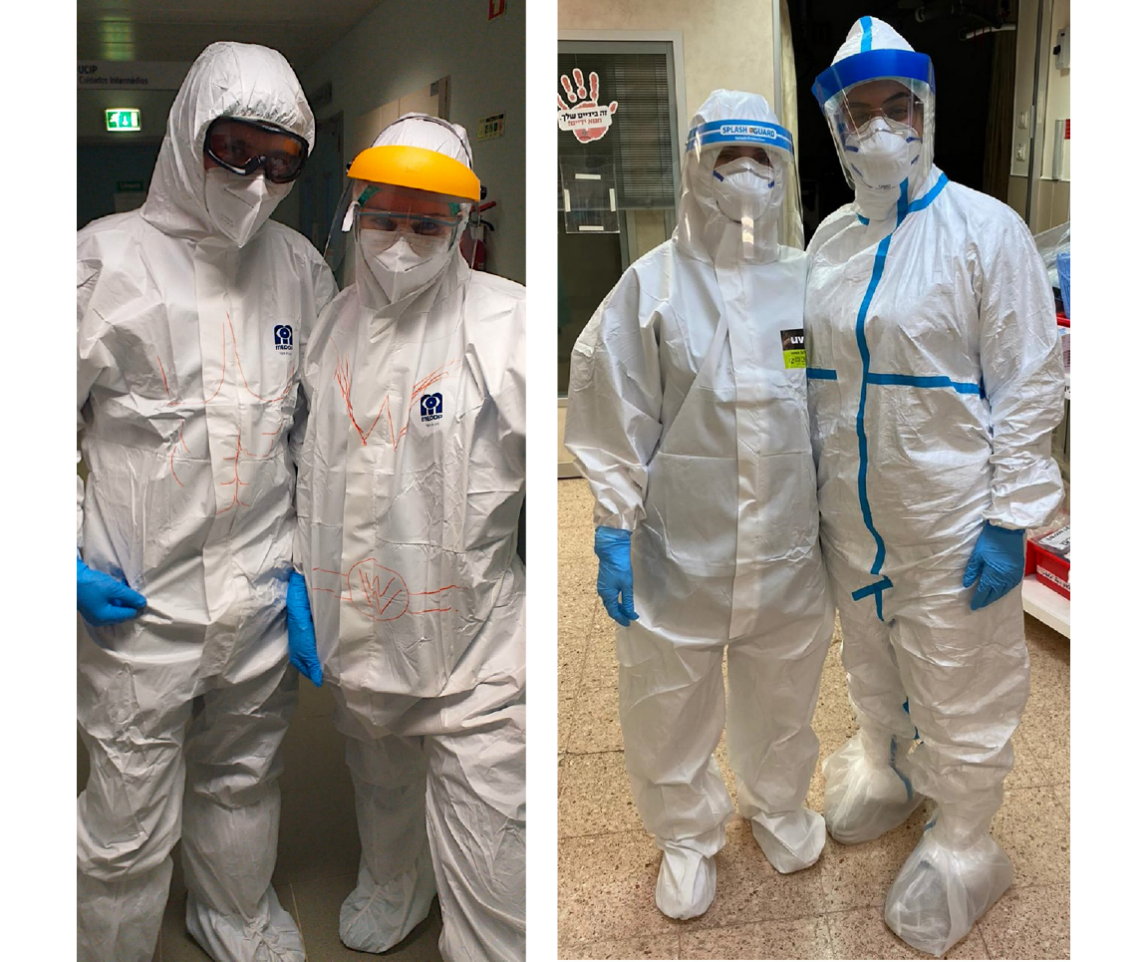
Typical level 1 personal protective equipment used in Portugal (left) and Israel (right).

Similar level 1 PPE is used in the two countries as is shown in Figure 1.

Table 1 presents the COVID-19 status in each country on May 1, 2020.

**Table 1:** Coronavirus disease summary statistics for Israel and Portugal, as of May 1, 2020.

Statistic	Israel	Portugal
Total confirmed cases, n	16,101	25,351
New cases^a^, n	58	306
Total deaths, n	225	1007
New deaths^a^, n	1	18
Total recovered, n	9156	1647
Active cases, n	7023	22,697
Intensive care unit cases, n	103	154
Total tests, n	390,022	395,771

^a^During the last 24 hours, as of May 1, 2020.

### Survey Design

The survey consisted of ten items relating to factors that have been found to have influence during the use of full PPE. The survey included general information about the objective of the study and a photo of a person wearing level 1 PPE.

Each item of the survey stated either the difficulty or ease of an influencing factor. Half the statements were worded as a difficulty and half as an easing influence so as to mitigate response bias. The statements were as follows in this order: (1) it is easy to put on (don) the full PPE; (2) wearing the full PPE is very uncomfortable; (3) it is hard to see everything around me while wearing the full PPE; (4) it is easy to hear sounds and speech around me while wearing the full PPE; (5) it is easy to understand what is said to me while wearing the full PPE; (6) it is easy to understand my surroundings while wearing the full PPE; (7) it is hard to think clearly while wearing the full PPE; (8) it is easy to make decisions while wearing the full PPE; (9) it is hard to remove (dof) the full PPE; and (10) it is very important to wear the full PPE.

Responses were according to a Likert scale ranging from 1, completely disagree, to 5, completely agree. The questionnaire included three demographic questions regarding gender, profession, and the frequency of using the full PPE. All statements and demographic questions were presented in Hebrew to participants in Israel and in Portuguese to participants in Portugal.

The survey was administered through the Qualtrics (Qualtrics International Inc) online platform. It was available for participants from April 12 in Israel and from April 16 in Portugal, to May 1, 2020. This platform ensured that there were no multiple entries from the same individual.

The study was approved by the Institutional Review Board of the Israel Institute of Technology, Haifa, Israel. Participants received a general introduction to the study and then presented with an informed consent form. In that form, participants were informed that the study does not pose any risk, that their participation is completely voluntary, and that they can quit at any time. In addition, they were informed that no personal data or identifying details were required nor collected, and that the data was secure and their participation remained anonymous. Participants had to select the “I Agree” option before entering the survey itself.

### Recruitment

We used a combination of criterion-based purposive and convenience sampling. The criterion for the purposive sampling was prehospital and hospital health care professionals involved in the care of patients with COVID-19 and using level 1 PPE. Using professional email lists and closed professional WhatsApp and Facebook groups, we recruited 1013 participants in Israel. In a similar fashion, we recruited 519 participants in Portugal.

### Statistical Analysis

#### Data Exclusion

Of the original 1013 persons who entered the survey in Israel, 189 did not proceed with responding and were thus excluded from the analyses. Of the original 519 who entered the survey in Portugal, 104 did not proceed with responding and were thus excluded from the analyses. We assume that many who consented to participate and entered the survey may have realized that the questions focused only on the use of level 1 PPE and elected not to continue. In addition, the goal of the study was to examine the influences of using the PPE based on extended and intensive use typical of the COVID-19 situation. Consequently, participants who reported rarely or never using the level 1 PPE may have responded to the questions based on their training or other sources but not necessarily based on personal experience and were thus excluded from the final survey analysis. In the Israeli sample, this amounted to 102 (12% of the participating respondents), and in the Portugal sample, this amounted to 114 (27% of the participating respondents).

#### Scoring the Responses

To achieve uniform direction of the responses on the 5-category Likert scale, the responses on items worded as a statement of ease were reversed. The objective was that all high responses would indicate greater difficulty and low responses would indicate greater ease. Specifically, the responses to item 1, 4, 5, 6, and 8 were reversed for the subsequent analyses.

## Results

### Respondent Statistics

A total of 722 respondents from Israel (of a total of 824 who participated) were included in the analysis. This sample consisted of 346 (48%) who reported using the level 1 PPE for at least a few hours daily and 376 (52%) who reported its use for at least a few hours weekly. This sample included 524 (72%) males and 198 (28%) females. In addition, 66 (9%) were physicians, 46 (6%) were from nursing professions, 299 (41%) were paramedics, 269 (37%) were medics, and 42 (7%) were of other occupations.

A total of 301 respondents from Portugal (of a total of 415 who participated) were included in the analysis. This sample consisted of 150 (49%) who reported using the level 1 PPE for at least a few hours daily and 151 (51%) who reported its use for at least a few hours weekly. This sample included 168 (55%) males and 133 (45%) females. In addition, 69 (24%) were physicians, 130 (43%) were from nursing professions (which in Portugal includes prehospital emergency work, parallel to the paramedics in Israel), 64 (21%) were medics, and 37 (12%) were of other occupations.

### Reliability

To assess reliability of the questionnaire items, a Cronbach α was computed for each of the samples. Cronbach α for the sample from Israel was .73 and, for the sample from Portugal, .75. This reliability index indicates an acceptable internal consistency of the responses.

### Overall Responses on the Questionnaire Items

Medians and their corresponding ranges were computed for each of the questionnaire items, stratified by health care profession, for each of the two countries. In addition, the percentage of respondents indicating an agreement and a strong agreement with a given questionnaire statement were computed. These percentages represented the proportion of respondents that expressed more difficulty with a given PPE factor. These statistics are presented in Table 2.

**Table 2:** Medians for each of the survey items, together with the proportions of respondents who agreed and strongly agreed with the difficulty, stratified by profession, for each of the countries.

Survey Item/Profession		Israel					Portugal			
		Score, median (range)		Participants, n/N (%)			Score, median (range)		Participants, n/N (%)	
**Difficulty of donning**										
	Medicine	2 (1-5)		18/67 (27)			3 (1-5)		53/96 (55)	
	Nursing	2 (1-5)		16/26 (38)			2 (1-5)		60/140 (43)	
	Paramedics	2 (1-5)		65/268 (24)			—^a^		—	
	Medics	2 (1-5)		76/264 (29)			2 (1-5)		27/74 (36)	
	Other	2 (1-5)		13/43 (30)			2.5 (1-5)		22/52 (42)	
**Discomfort**										
	Medicine	4 (1-5)		42/61 (69)			4 (1-5)		96/102 (94)	
	Nursing	4 (1-5)		32/45 (71)			4 (1-5)		133/150 (89)	
	Paramedics	4 (1-5)		224/264 (85)			—		—	
	Medics	4 (1-5)		208/280 (74)			4 (1-5)		39/51 (82)	
	Other	4 (1-5)		33/38 (87)			4 (1-5)		39/51 (76)	
**Difficulty in seeing**										
	Medicine	4 (2-5)		55/70 (78)			4 (1-5)		82/94 (87)	
	Nursing	4 (2-5)		38/44 (86)			4 (1-5)		139/153 (91)	
	Paramedics	4 (2-5)		280/300 (93)			—		—	
	Medics	4 (1-5)		266/303 (88)			4 (1-5)		55/73 (75)	
	Other	4 (1-5)		40/46 (87)			3.5 (1-5)		40/50 (77)	
**Difficulty in hearing**										
	Medicine	3 (1-5)	31/65 (48)			4 (1-5)		68/97 (70)		
	Nursing	4 (1-5)	27/39 (69)			4 (1-5)		105/146 (72)		
	Paramedics	3 (1-5)	132/262 (50)			—		—		
	Medics	3 (1-5)	100/259 (38)			3 (1-5)		37/75 (49)		
	Other	3 (1-5)	24/36 (67)			3 (1-5)		25/49 (51)		
**Difficulty in understanding speech**										
	Medicine	3 (1-5)	32/61 (52)			4 (1-5)		70/96 (73)		
	Nursing	4 (1-5)	28/37 (76)			4 (1-5)		109/144 (76)		
	Paramedics	3 (1-5)	135/251 (54)			—		—		
	Medics	3 (1-5)	106/258 (41)			3 (1-5)		34/75 (45)		
	Other	3 (1-5)	20/34 (59)			3 (1-5)		29/53 (55)		
**Difficulty in understanding the surroundings**										
	Medicine	3 (1-5)	25/52 (48)			4 (1-5)		73/96 (76)		
	Nursing	3 (1-5)	24/39 (61)			4 (1-5)		107/147 (73)		
	Paramedics	3 (1-5)	147/236 (62)			—		—		
	Medics	3 (1-5)	108/238 (45)			4 (1-5)		45/72 (62)		
	Other	3 (1-5)	21/34 (62)			3 (1-5)		26/48 (54)		
**Difficulty in thinking clearly**										
	Medicine	2 (1-5)			22/60 (37)		4 (1-5)			50/84 (59)
	Nursing	3 (1-5)			24/41 (58)		4 (1-5)			84/123 (68)
	Paramedics	3 (1-5)			121/245 (49)		—			—
	Medics	2 (1-5)			92/260 (35)		3 (1-5)			27/64 (42)
	Other	3 (1-5)			16/38 (42)		2 (1-5)			19/48 (40)
**Difficulty in making decisions**										
	Medicine	2.5 (1-5)			14/51 (27)		3 (1-5)			50/79 (63)
	Nursing	3 (1-5)			15/33 (45)		3 (1-5)			64/119 (54)
	Paramedics	3 (1-5)			107/208 (51)		—			—
	Medics	3 (1-5)			69/226 (30)		2 (1-5)			25/67 (37)
	Other	3 (1-5)			13/34 (38)		2.5 (1-5)			18/48 (37)
**Difficulty of doffing**										
	Medicine	3 (1-5)			38/70 (54)		4 (1-5)			80/93 (86)
	Nursing	4 (2-5)			25/44 (57)		4 (1-5)			121/149 (81)
	Paramedics	3 (1-5)			124/277 (45)		—			—
	Medics	2 (1-5)			110/280 (40)		4 (1-5)			54/76 (71)
	Other	2.5 (1-5)			18/42 (43)		4 (1-5)			34/52 (65)
**The importance of wearing the personal protective equipment**										
	Medicine	5 (2-5)			74/75 (99)		5 (4-5)			104/107 (97)
	Nursing	5 (2-5)			47/48 (98)		5 (4-5)			159/160 (99)
	Paramedics	5 (1-5)			294/299 (98)		—			—
	Medics	5 (1-5)			314/319 (98)		5 (4-5)			84/84 (100)
	Other	5 (3-5)			46/46 (100)		5 (3-5)			60/60 (100)

^a^The emergency medical services in Portugal do not include paramedics; nurses working in prehospital settings fulfill parallel functions.

Responses to items relating to the physical and ergonomic aspects showed high agreement across professions and in both countries that the use of the PPE is highly uncomfortable: 78% (n=539/688) in Israel and 87% (n=328/377) in Portugal. Only 27% (n=188/684) of the respondents from Israel and 45% (n=163/365) from Portugal, of all the professions, indicated that donning the PPE was difficult. Agreement was high across professions and the two countries regarding difficulty in seeing what is going on around while using the PPE: 89% (n=697/763) in Israel and 84% (n=317/376) in Portugal. Finally, almost all the respondents of all the professions, 99% in both countries, expressed the importance of using the PPE.

Significantly higher proportions of respondents from Portugal than Israel reported difficulties in doffing the PPE: 77% (n=290/374) vs 44% (n=315/713; χ^2^_1_=110, *P*<.001); in hearing: 64% (n=236/370) vs 50% (n=321/641; χ^2^_1_=25.2, *P*<.001); in understanding speech: 65% (n=243/372) vs 47% (n=314/661; χ^2^_1_=22.1, *P*<.001); in understanding the situation: 69% (n=253/367) vs 54% (n=325/599; χ^2^_1_=20.4, *P*<.001); in being able to think clearly: 57% (n=183/323) vs 43% (n=275/644; χ^2^_1_=16.8, *P*<.001); and in being able to make decisions: 50% (n=158/317) vs 39% (n=218/552; χ^2^_1_=8.8, *P*<.001).

### Discovery of Common Factors

When variables of a certain set correlate with each other, a certain factor may exist that correlates with the set of variables. To discover such possible underlying factors in the data, an exploratory factor analysis (EFA) was performed to reduce the data set into a smaller number of variables. Factor analysis identifies factors that may explain correlations among variables. Accordingly, each variable has a relative weight, or loading, within a given factor. Statistical indices of the analysis indicate that our data were suitable for factor analysis and suitable for the specific factor analysis reduction technique. The detailed statistics of the factor analysis for each country are included in Multimedia Appendix 1 for Israel and Multimedia Appendix 2 for Portugal.

The factor analysis uncovered two factors, which are presented for both countries in Table 3. For both countries, the first factor showed high loadings for three questionnaire items: difficulty in hearing, difficulty in understanding speech, and difficulty in understanding the situation. This factor suggests that when the full PPE is on, increased difficulties in hearing and understanding speech imply difficulties in spoken communication, which were related to difficulties in understanding the surroundings, that is, situational awareness.

**Table 3:** Relative weights (loading coefficients) of all the questionnaire items according to the two factors identified for each country^a^.

Questionnaire items	Israel			Portugal	
	Factor 1	Factor 2	Factor 1		Factor 2
Donning	0.05	*0.65* ^b^	0.29		0.53
Discomfort	0.33	0.56	0.19		*0.70*
Seeing	0.40	0.43	0.07		*0.71*
Hearing	*0.87*	0.02	*0.84*		0.07
Understanding speech	*0.89*	0.04	*0.87*		0.16
Understanding the surroundings	*0.64*	0.36	*0.80*		0.13
Thinking clearly	0.11	*0.63*	0.10		0.55
Making decisions	0.42	0.45	0.44		0.18
Doffing	0.02	*0.61*	0.06		*0.67*

^a^The analysis did not include the last item of the questionnaire, regarding the significance of having the personal protective equipment, since there was little variation in the responses to that question.

^b^Relative weight values higher than 0.6 are in italics.

Less similarity was observed between the two countries in the second factor. The Israeli data show loadings slightly higher than 0.6 for the items of difficulty of donning the PPE, difficulty in thinking clearly, and difficulty of doffing, with discomfort being close to a loading of 0.6. The Portuguese sample showed loadings higher than 0.6 for the items of discomfort, difficulty in seeing, and difficulty of doffing. Taken together, this factor implies a construct relating to the physical aspects of having the PPE, primarily related to doffing, discomfort, and donning. Interestingly, the second factor in the Portuguese sample includes the item of difficulty in seeing.

### PPE Discomfort, Communication, and Situational Awareness: Relations Between the Constructs

**Figure 2:**
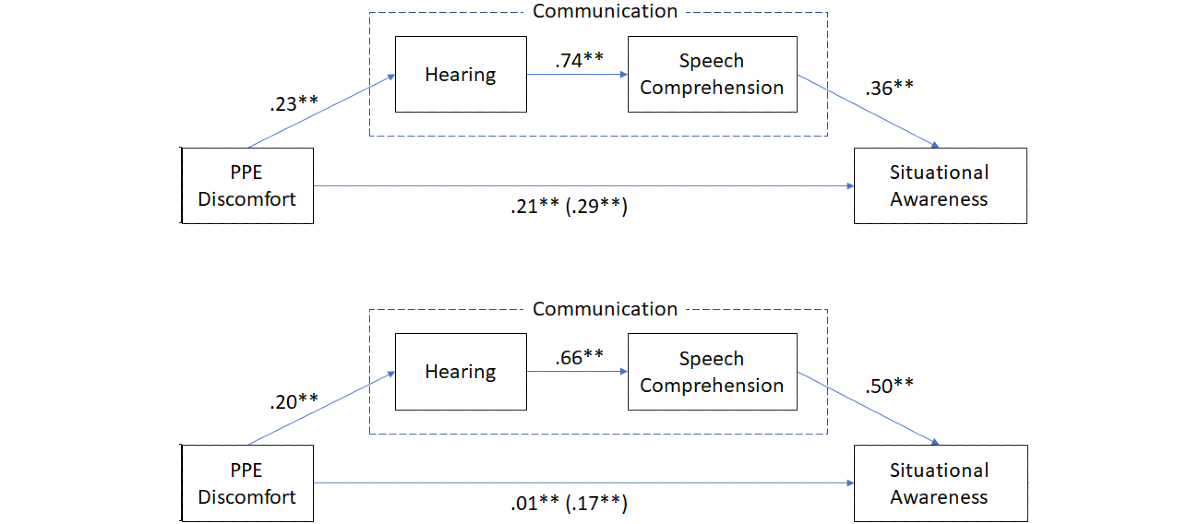
Mediation models of the responses in Israel (top model) and Portugal (bottom model). Each link is associated with its corresponding standardized coefficient, together with the significance indication (** indicates a significance level of *P*<.01). The direct link between PPE discomfort and situational awareness shows the total effect, including the presence of the mediating variables; the direct effect without the mediating factors is in parenthesis. PPE: personal protective equipment.

We further explored the possibility that wearing the PPE influences communication, as manifested through difficulties in hearing and understanding speech, which in turn influences situational awareness. Specifically, we examined the extent that discomfort influenced communication (ie, hearing and understanding speech), which in turn influenced situational awareness. We employed a mediation analysis based on [33], using the Process Macro in SPSS v21 (IBM Corp). Specifically, we used model 6, which assumes two mediators between an independent variable and the outcome. In this study, the extent of difficulties in hearing while having the PPE on was the independent variable, and the outcome was the extent that one understands the surroundings. We explored the extent of hearing and understanding what is spoken while wearing the PPE, as possible mediating variables in the influence of PPE discomfort on situational awareness. This mediation analysis was performed on the data of each country. Both mediation analyses are presented in Figure 2.

As shown in Figure 2, discomfort had a significant direct effect on situational awareness, as evident by the significant coefficients in parenthesis, for both countries. This effect decreased significantly when mediation by hearing and understanding speech was considered. This implies a significant indirect effect of discomfort on situational awareness, as mediated by hearing and understanding speech. Increased discomfort with the PPE was related to increased difficulties in hearing and speech comprehension, which were related to increased difficulties in understanding the surroundings. According to this analysis, the mediating variables of hearing and understanding speech could account for 31% of the total effect of discomfort on situational awareness in Israel and 94% in Portugal. Although the effect size in the Portuguese data was much stronger than in the Israeli data, the same relations were demonstrated significantly in both countries.

## Discussion

### Principal Results

During the period this study was conducted, level 1 PPE was used extensively to mitigate contamination of health care professions treating patients with suspected or confirmed COVID-19. A survey of health care workers in two countries, Israel and Portugal, showed that the use of level 1 PPE is uncomfortable and entails difficulties in removing (doffing) it. Moreover, the discomfort appeared to be associated with difficulties in perceptual and cognitive functioning. Specifically, the equipment was found to be associated with difficulties in visual and auditory perception, and difficulties in understanding speech, situational awareness, and thought and decision making.

The findings of the survey uncovered strong relations between difficulties in auditory perception, the ability to understand speech, and situational awareness. Auditory perception and the ability to understand what is being said are fundamental and critical to speech communication. Communication is critical to teamwork [34] and to situational awareness [35]. Additional analysis of the survey responses shows associations of increased discomfort with the PPE with greater difficulties in communication, namely hearing and understanding speech; these in turn were associated with degraded situational awareness.

The mediation of communication in the influence of PPE discomfort on situational awareness has both interesting theoretical and practical implications. Theoretically, this implies that perceptual and cognitive processes play a significant role in the influence of a physical factor, discomfort in this case, and on a cognitive factor, situational awareness in this case. However, beyond these theoretical implications, practical implications can and should be drawn from such findings.

### Practical Implications

Effective design of PPE and adequate sequence and training of donning and doffing are aimed at two major objectives: (1) protect health care workers from contamination; and (2) minimize interference with performing medical and nursing tasks. This section discusses practical implications of the similarities and differences in the responses between the two countries in terms of PPE design, donning and doffing procedures, training, and culture. It also addresses the findings in terms of short- and long-term implementations.

The results showed overall agreement across professions and the two countries concerning the discomfort of the PPE. Accounting for these similar responses is the fact that the components of the PPE, and the steps and sequences of donning and doffing PPE are similar for Israel and Portugal, with both following the CDC and WHO recommendations [36]. The donning sequence is: (1) gown or coverall; (2) mask or respirator; (3) goggles; (4) face shield; and (5) gloves. The doffing sequence is the reverse. Improper use of PPE is known to spread infection among health care workers [4,5]. All these steps can be sources of errors. This is especially true in the context of long work shifts and harsh environmental settings, as the design of PPE materials does not yet account for user temperature and hydration homeostasis. Both reusable and disposable PPE should be clearly designated such as with color coding and designed for easy donning and doffing [37,38]. The simpler the system (with fewer parts), the more likely it will avoid human distractions that may lead to contamination. Regarding contamination protection, although PPE is often only worn for short periods of time, pathogenic viruses such as influenza, severe acute respiratory syndrome, and Ebola can survive for extended periods of time on surfaces and be sources of transmission via surface-to-hand and hand-to-face or -mucous membrane contact. Despite two layers of protective clothing and sometimes two pairs of gloves, hand hygiene remains an essential aspect of PPE.

Full-face covers can provide adequate mucous membrane protection, such as the nasal cavities, lips, and mouth [39]. Whether by using positive pressure systems or only barrier filters, we believe that full-face integrated systems may widen the field-of-view and offer advantages for situational awareness. Such system should be designed to minimize condensation. In addition, a single system is simpler to maintain and minimizes efforts in donning and doffing.

Much of the research on influences of using PPE has focused on influences of the equipment on the motoric aspects of user performance [27,29,40]. The findings here suggest that the use of level 1 PPE also has perceptual and cognitive influences. Relating to communication and situational awareness, some key technological directions and advancements can be considered, such as audio and communication-enhancing technologies. These should be hands-free technologies under the PPE, such as headsets and microphones or throat microphones based on Bluetooth, Wi-Fi, or radio ultra-high frequency. These can be interfaced with dedicated mobile phone apps and facilitate effective hearing and talking among health care workers.

Longer-term consideration of technologies that promote more effective use of level 1 PPE and that facilitate perceptual and cognitive functioning can include:

- Virtual reality technology: the use of virtual reality for training purposes, such as was previously proposed [41], can facilitate training in effective communication, teamwork, and situational awareness during the use of the equipment.
- Augmented reality technology: this single key aspect may boost the ergonomics of a full-face system and act synergistically when connected with a hands-free voice-activated communication device.
- Internet of Things (IoT): capable 5G IoT devices coupled with mesh framework solutions and cheap microcomputers (or even smartphones) can contribute to a solution. Ultrafast transmission rates and processing power, allied with powerful cloud servers, will eliminate bandwidth problems and eventually clear the way for artificial intelligence “Wingmen.” This should help overcome the previously mentioned limitations in PPE while providing advanced advice immediately [42].

The responses from Portugal reflected more difficulties in hearing, understanding speech, and understanding the situation in addition to more difficulties in thinking clearly and making decisions compared with the responses from Israel. These differences could be accounted for by the differences in training and overall culture. Specifically, the prehospital professionals in Israel, paramedics and medics, undergo more extensive training in using PPE as an overall preparedness to various chemical, biological, radiological, nuclear, and explosive materials incidents. This is in addition to the training at the breakout of the COVID-19 crisis. In contrast, the health care professionals in Portugal received training only when the crisis started. Thus, beyond considering the redesign of level 1 PPE and implementation of newer technologies to facilitate visual and auditory perception, more periodic training and procedures related to cognitive functioning can be implemented immediately. These can include practicing and adopting visual scanning patterns, and communication protocols with coworkers to facilitate teamwork situational awareness.

Another factor that could account for some of the differences between the countries are cultural and overall political considerations. Israel thus far is in a constant state of preparedness to multi-casualty incidents, and consequently health care professionals are in a different mindset when encountering a crisis compared with other countries. Thus, when dealing with a global crisis such as the COVID-19 pandemic, cultural and political characteristics should be considered.

### Limitations and Future Research

The research has some limitations that should be addressed in follow-up and future studies. First, the definition of a paramedic differed between the two countries. In Israel, paramedics are all prehospital emergency workers; in Portugal, the nursing profession includes prehospital and in-hospital emergency workers. This distinction was not captured in the data and analyses. Second, the sampling method was indeed purposive but also comprised convenience sampling through the professional networks in each of the countries. This sampling approach may not have included other health care workers who used level 1 PPE during the pandemic, such as community health care workers. Such data would have enriched the data, findings, and conclusions. Third, the survey instructions specified the details of level 1 PPE, including a photo to visualize it. Nevertheless, we acknowledge the availability of a variety of level 1 PPE in the world, of different brands and including different components. The exact PPE used by each respondent was not captured in the data and should be documented and analyzed in future studies.

Future studies should include simulation-based experiments in which various PPE designs and procedures can be compared in terms of their influence on potential contamination and on physical, perceptual, and cognitive functioning. In addition, analytic techniques such as failure mode and effects analysis along with the risk priority ratings should accompany empirical studies.

### Conclusions

From 2014 to 2016, a global epidemic of the Ebola virus made proper use of PPE a paramount concern in health care settings [43]. Nevertheless, almost no innovations were implemented in terms of PPE ergonomics to address limitations in hearing, vision, or even comfort. The COVID-19 pandemic serves as a “field test” for PPE technology, as every health care system in the world must adapt its use on a daily basis. Our binational study found that health care workers from different countries and cultures, yet similar NHS and PPE level approaches, shared the same difficulties and felt their situational awareness undermined by outdated PPE designs.

In 2020, the COVID-19 pandemic is paving the way for updating PPE design. The use of already deployed technology provides abundant opportunities to improve, adapt, and overcome problems. This should be done in a practical and aesthetically pleasing way, as well as appropriately to endure future epidemics of this century.

## Appendix

Multimedia Appendix 1 Factor analysis statistics for Israel.

Multimedia Appendix 2 Factor analysis statistics for Portugal.

## Abbreviations

COVID-19: coronavirus disease
EFA: exploratory factor analysis
EMT: emergency medical technician
IoT: Internet of Things
NHS: national health care system
PPE: personal protective equipment

## References

[1] (2020). World Health Organization.

[2] Akbar-Khanzadeh F, Bisesi MS, Rivas RD (1995). Comfort of personal protective equipment. Appl Ergonomics.

[3] Mumma J, Durso F, Casanova L, Erukunuakpor K, Kraft CS, Ray SM, Shane AL, Walsh VL, Shah PY, Zimring C, DuBose J, Jacob JT (2019). Common behaviors and faults when doffing personal protective equipment for patients with serious communicable diseases. Clin Infect Dis.

[4] Hallihan G, Baers J, Wiley K, Davies J, Kaufman J, Conly J, Caird J (2015). Human factors evaluation to identify systems factors to improve safety during donning and doffing personal protective equipment (PPE) in Ebola virus disease management scenario. Open Forum Infect Dis.

[5] Gurses AP, Dietz AS, Nowakowski E, Andonian J, Schiffhauer M, Billman C, Abashian AM, Trexler P, Osei P, Benishek LE, Xie A, Pronovost P, Rosen MA, Maragakis LL, CDC Prevention Epicenter Program (2019). Human factors-based risk analysis to improve the safety of doffing enhanced personal protective equipment. Infect Control Hosp Epidemiol.

[6] Akbar-Khanzadeh F (1998). Factors contributing to discomfort or dissatisfaction as a result of wearing personal protective equipment. J Human Ergol.

[7] de Almeida RACDS, Veiga MM, de Castro Moura Duarte FJ, Meirelles LA, Veiga LBE (2012). Thermal comfort and personal protective equipment (PPE). Work.

[8] Grugle NL, Kleiner BM (2007). Effects of chemical protective equipment on team process performance in small unit rescue operations. Appl Ergon.

[9] Rebmann T, Carrico R, Wang J (2013). Physiologic and other effects and compliance with long-term respirator use among medical intensive care unit nurses. Am J Infect Control.

[10] John A, Tomas ME, Cadnum JL, Mana TS, Jencson A, Shaikh A, Zabarsky TF, Wilson BM, Donskey CJ (2016). Are health care personnel trained in correct use of personal protective equipment?. Am J Infect Control.

[11] Kwon JH, Burnham CD, Reske KA, Liang SY, Hink T, Wallace MA, Shupe A, Seiler S, Cass C, Fraser VJ, Dubberke ER (2017). Assessment of healthcare worker protocol deviations and self-contamination during personal protective equipment donning and doffing. Infect Control Hosp Epidemiol.

[12] Nichol K, McGeer A, Bigelow P, O'Brien-Pallas L, Scott J, Holness DL (2013). Behind the mask: determinants of nurse's adherence to facial protective equipment. Am J Infect Control.

[13] Coates M, Jundi A, James M (2000). Chemical protective clothing; a study into the ability of staff to perform lifesaving procedures. J Accid Emerg Med.

[14] Cavazza N, Serpe A (2009). Effects of safety climate on safety norm violations: exploring the mediating role of attitudinal ambivalence toward personal protective equipment. J Safety Res.

[15] Olson R, Grosshuesch A, Schmidt S, Gray M, Wipfli B (2009). Observational learning and workplace safety: the effects of viewing the collective behavior of multiple social models on the use of personal protective equipment. J Safety Res.

[16] Neves HCC, Souza ACSE, Medeiros M, Munari DB, Ribeiro LCM, Tipple AFV (2011). Safety of nursing staff and determinants of adherence to personal protective equipment. Rev Lat Am Enfermagem.

[17] AlGhamri AA, Murray SL, Samaranayake VA (2013). The effects of wearing respirators on human fine motor, visual, and cognitive performance. Ergonomics.

[18] Gaston JR, Mermagen T, Foots A, Dickerson K (2015). Auditory localization performance in the azimuth for tactical communication and protection systems. J Acoust Soc Am.

[19] Scharine A, Letowski T, Sampson JB (2009). Auditory situation awareness in urban operations. J Milit Strategic Stud.

[20] Wade L, Weimar W, Davis J (2004). Effect of personal protective eyewear on postural stability. Ergonomics.

[21] Brown MN, Char RMML, Henry SO, Tanigawa J, Yasui S (2019). The effect of firefighter personal protective equipment on static and dynamic balance. Ergonomics.

[22] Wade C, Davis J, Marzilli TS, Weimar WH (2006). Information processing capacity while wearing personal protective eyewear. Ergonomics.

[23] Park J (2019). The adverse impact of personal protective equipment on firefighters’ cognitive functioning. Res J Costumec.

[24] Gupta D, Kumar S (2018). WebmedCentral.com.

[25] Salehi H, Pennathur PR, Da Silva JP, Herwaldt LA (2019). Examining health care personal protective equipment use through a human factors engineering and product design lens. Am J Infect Control.

[26] Kwon JH, Burnham CD, Reske KA, Liang SY, Hink T, Wallace MA, Shupe A, Seiler S, Cass C, Fraser VJ, Dubberke ER (2017). Assessment of healthcare worker protocol deviations and self-contamination during personal protective equipment donning and doffing. Infect Control Hosp Epidemiol.

[27] Castle N, Bowen J, Spencer N (2010). Does wearing CBRN-PPE adversely affect the ability for clinicians to accurately, safely, and speedily draw up drugs?. Clin Toxicol (Phila).

[28] Wiyor HD, Coburn JC, Siegel KL (2020). Impact of clinician personal protective equipment on medical device use during public health emergency: a review. Disaster Med Public Health Prep.

[29] Johnson AT (2016). Respirator masks protect health but impact performance: a review. J Biol Eng.

[30] Dayan M, Ward D, Gardner T, Kelly E (2018). The Health Foundation.

[31] Central Bureau of Statistics.

[32] Central Intelligence Agency.

[33] Hayes A (2017). Introduction to Mediation, Moderation, and Conditional Process Analysis: A Regression-Based Approach.

[34] Gluyas H (2015). Effective communication and teamwork promotes patient safety. Nurs Stand.

[35] Parush A, Kramer C, Foster-Hunt T, Momtahan K, Hunter A, Sohmer B (2011). Communication and team situation awareness in the OR: implications for augmentative information display. J Biomed Inform.

[36] Centers for Disease Control and Prevention.

[37] Reddy S, Valderrama A, Kuhar D (2019). Improving the use of personal protective equipment: applying lessons learned. Clin Infect Dis.

[38] Drews F, Mulvey D, Stratford K, Samore M, Mayer J (2019). Evaluation of a redesigned personal protective equipment gown. Clin Infect Dis.

[39] Yan Y, Chen H, Chen L, Cheng B, Diao P, Dong L, Gao X, Gu H, He L, Ji C, Jin H, Lai W, Lei T, Li L, Li L, Li R, Liu D, Liu W, Lu Q, Shi Y, Song J, Tao J, Wang B, Wang G, Wu Y, Xiang L, Xie J, Xu J, Yao Z, Zhang F, Zhang J, Zhong S, Li H, Li H (2020). Consensus of Chinese experts on protection of skin and mucous membrane barrier for health professions fighting against coronavirus disease 2019. Chin J Dermatol.

[40] Loibner M, Hagauer S, Schwantzer G, Berghold A, Zatloukal K (2019). Limiting factors for wearing personal protective equipment (PPE) in a health care environment evaluated in a randomised study. PLoS One.

[41] Ragazzoni L, Ingrassia PL, Echeverri L, Maccapani F, Berryman L, Burkle FM, Della Corte F (2015). Virtual reality simulation training for Ebola deployment. Disaster Med Public Health Prep.

[42] Podgórski D, Majchrzycka K, Dąbrowska A, Gralewicz G, Okrasa M (2017). Towards a conceptual framework of OSH risk management in smart working environments based on smart PPE, ambient intelligence and the Internet of Things technologies. Int J Occup Saf Ergon.

[43] Centers for Disease Control and Prevention.

