# Supplementary material for: Human Factor Considerations in Using Personal Protective Equipment in the COVID-19 Pandemic Context: Binational Survey Study
Source: J Med Internet Res. 2020 Jun 17;22(6):e19947. doi: 10.2196/19947 (PMC7301688; doi:10.2196/19947)
Supplement: Multimedia Appendix 1 [file jmir_v22i6e19947_app1.pdf]

Appendix A – Factor Analysis Statistics for Israel

Table A-1. Correlation Matrix

Variable		Donning	Discomfort	Vision	Hearing	Speech	SA	Thinking	Decisions	Doffing
**Correlation**										
	Donning	1.000	.233	.128	.120	.155	.229	.243	.229	.398
	Discomfort	.233	1.000	.414	.231	.218	.303	.330	.260	.155
	Vision	.128	.414	1.000	.221	.241	.410	.221	.228	.108
	Hearing	.120	.231	.221	1.000	.751	.399	.112	.241	.093
	Speech	.155	.218	.241	.751	1.000	.465	.154	.343	.123
	SA	.229	.303	.410	.399	.465	1.000	.263	.365	.154
	Thinking	.243	.330	.221	.112	.154	.263	1.000	.318	.162
	Decisions	.229	.260	.228	.241	.343	.365	.318	1.000	.158
	Doffing	.398	.155	.108	.093	.123	.154	.162	.158	1.000
**Sig. (1-tailed)**										
	Donning		.000	.000	.000	.000	.000	.000	.000	.000
	Discomfort	.000		.000	.000	.000	.000	.000	.000	.000
	Vision	.000	.000		.000	.000	.000	.000	.000	.001
	Hearing	.000	.000	.000		.000	.000	.001	.000	.004
	Speech	.000	.000	.000	.000		.000	.000	.000	.000
	SA	.000	.000	.000	.000	.000		.000	.000	.000
	Thinking	.000	.000	.000	.001	.000	.000		.000	.000
	Decisions	.000	.000	.000	.000	.000	.000	.000		.000
	Doffing	.000	.000	.001	.004	.000	.000	.000	.000	

SA: Situational awareness

Table A-2. KMO and Bartlett's Test

Statistical test		Value
**Kaiser-Meyer-Olkin Measure of Sampling adequacy**		.742
**Bartlett's Test of Sphericity**		
	Approx. Chi-Square	1816.597
	df	36
	Sig.	.000

Table A-3. Communalities

Variable	Initial	Extraction
Donning	1.000	.495
Discomfort	1.000	.405
Vision	1.000	.330
Hearing	1.000	.749
Speech	1.000	.780
SA	1.000	.541
Thinking	1.000	.405
Decisions	1.000	.373
Doffing	1.000	.398

Extraction Method: Principal Component Analysis.
SA: Situational awareness

Table A-4. Total Variance Explained

Component	Initial Eigenvalues			Extraction Sums of Squared Loadings			Rotation Sums of Squared Loadings		
	Total	% of Variance	Cumulative %	Total	% of Variance	Cumulative %	Total	% of Variance	Cumulative %
1	3.106	34.510	34.510	3.106	34.510	34.510	2.438	27.084	27.084
2	1.370	15.225	49.735	1.370	15.225	49.735	2.039	22.651	49.735
3	1.080	12.001	61.736						
4	.840	9.335	71.071						
5	.689	7.661	78.732						
6	.603	6.696	85.428						
7	.595	6.609	92.036						
8	.479	5.327	97.364						
9	.237	2.636	100.000						

Extraction Method: Principal Component Analysis.

Figure A-1. A Scree Plot that shows the drastic decrease in the size of the Eigenvalues and helps determine the number of factors that accounts for most of the variation in the data

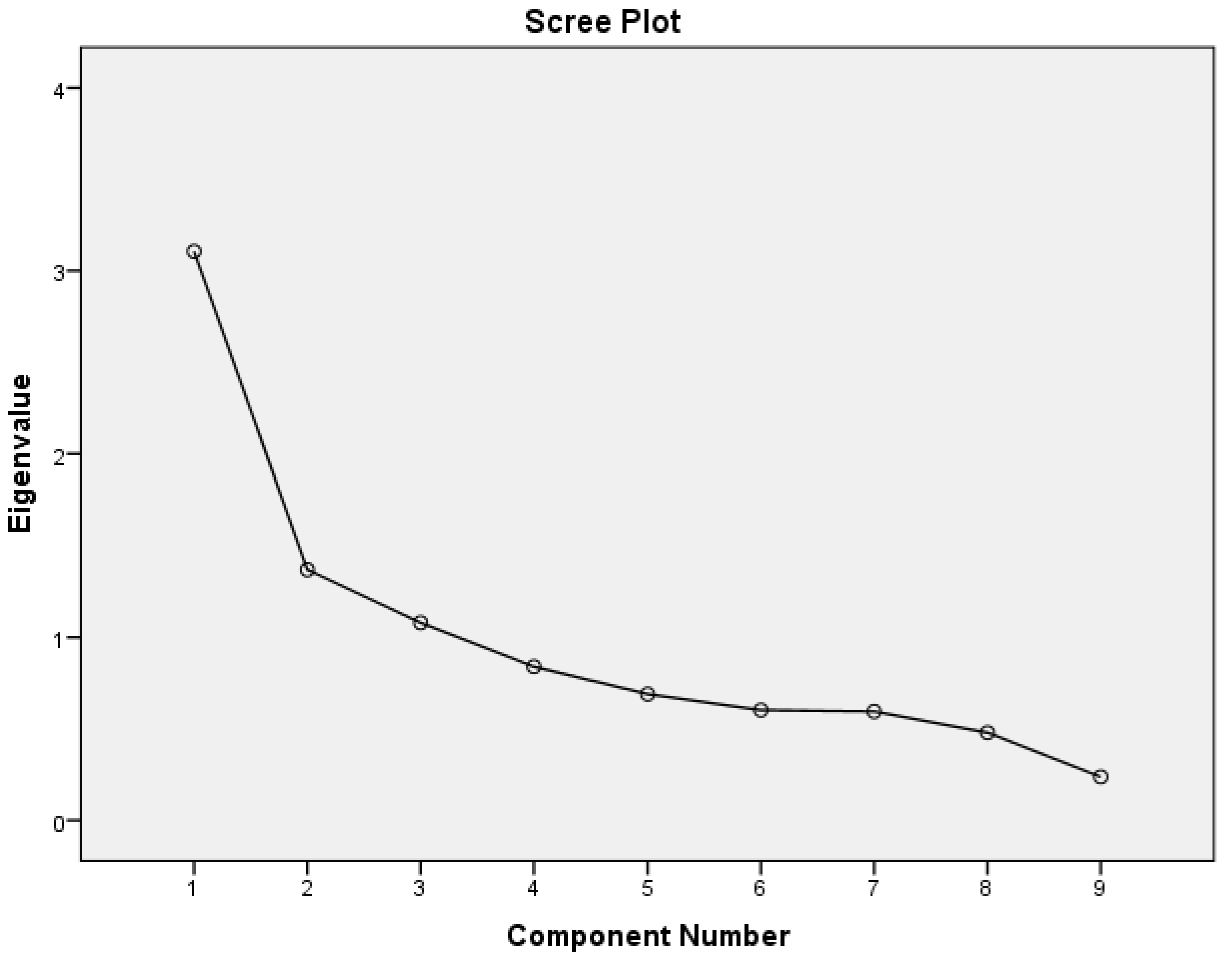

Table A-5. Initial Factor Matrix[a]

Variable	Component	
	1	2
Donning	.462	.530
Discomfort	.594	.230
Vision	.573	.044
Hearing	.655	-.567
Speech	.711	-.524
SA	.723	-.134
thinking	.503	.389
decisions	.605	.082
doffing	.367	.513

Extraction Method: Principal Component Analysis.

2 components extracted.

SA: Situational awareness

Table A-6. Rotated Component Matrix[a]

Variable	Component	
	1	2
Donning	.034	.703
Discomfort	.323	.549
Vision	.422	.390
Hearing	.865	-.038
Speech	.883	.030
SA	.650	.343
thinking	.153	.617
decisions	.424	.440
doffing	-.030	.630

Extraction Method: Principal Component Analysis.

Rotation Method: Varimax with Kaiser Normalization.[a]

Rotation converged in 3 iterations.

SA: Situational awareness

This table shows the convergent and discriminant validity:

Table A-7. Component Transformation Matrix

Component	1	2
1	.784	.621
2	-.621	.784

Extraction Method: Principal Component Analysis.

Rotation Method: Varimax with Kaiser Normalizatio
