# Supplementary material for: Human Factor Considerations in Using Personal Protective Equipment in the COVID-19 Pandemic Context: Binational Survey Study
Source: J Med Internet Res. 2020 Jun 17;22(6):e19947. doi: 10.2196/19947 (PMC7301688; doi:10.2196/19947)
Supplement: Multimedia Appendix 2 [file jmir_v22i6e19947_app2.pdf]

Appendix B – Factor Analysis Statistics for Portugal

Table B-1. Correlation Matrix

Variable		Donning	Discomfort	Vision	Hearing	Speech	SA	Thinking	Decisions	Doffing
**Correlation**										
	donning	1.000	.229	.126	.227	.263	.242	.139	.186	.328
	discomfort	.229	1.000	.405	.208	.257	.175	.267	.122	.329
	vision	.126	.405	1.000	.118	.230	.258	.232	.165	.279
	hearing	.227	.208	.118	1.000	.678	.490	.117	.263	.144
	speech	.263	.257	.230	.678	1.000	.605	.133	.274	.204
	SA	.242	.175	.258	.490	.605	1.000	.170	.331	.143
	thinking	.139	.267	.232	.117	.133	.170	1.000	.176	.181
	decisions	.186	.122	.165	.263	.274	.331	.176	1.000	.068
	doffing	.328	.329	.279	.144	.204	.143	.181	.068	1.000
**Sig. (1-tailed)**										
	donning		.000	.005	.000	.000	.000	.002	.000	.000
	discomfort	.000		.000	.000	.000	.000	.000	.006	.000
	vision	.005	.000		.008	.000	.000	.000	.000	.000
	hearing	.000	.000	.008		.000	.000	.009	.000	.002
	speech	.000	.000	.000	.000		.000	.003	.000	.000
	SA	.000	.000	.000	.000	.000		.000	.000	.002
	thinking	.002	.000	.000	.009	.003	.000		.000	.000
	decisions	.000	.006	.000	.000	.000	.000	.000		.084
	doffing	.000	.000	.000	.002	.000	.002	.000	.084	

SA: Situational awareness

Table B-2. KMO and Bartlett's Test

Statistical test		Value
**Kaiser-Meyer-Olkin Measure of Sampling Adequacy**		.764
**Bartlett's Test of Sphericity**		
	Approx. Chi-Square	822.455
	df	36
	Sig.	.000

Table B-3. Communalities

Variable	Initial	Extraction
Donning	1.000	.278
Discomfort	1.000	.555
Vision	1.000	.466
Hearing	1.000	.688
Speech	1.000	.753
SA	1.000	.644
Thinking	1.000	.302
Decisions	1.000	.273
Doffing	1.000	.471

Extraction Method: Principal Component Analysis.

SA: Situational awareness

Table B-4. Total Variance Explained

Component	Initial Eigenvalues			Extraction Sums of Squared Loadings			Rotation Sums of Squared Loadings		
	Total	% of Variance	Cumulative %	Total	% of Variance	Cumulative %	Total	% of Variance	Cumulative %
1	3.030	33.662	33.662	3.030	33.662	33.662	2.424	26.934	26.934
2	1.401	15.561	49.223	1.401	15.561	49.223	2.006	22.290	49.223
3	.970	10.777	60.001						
4	.879	9.761	69.762						
5	.760	8.440	78.202						
6	.623	6.927	85.129						
7	.598	6.649	91.778						
8	.448	4.981	96.759						
9	.292	3.241	100.000						

Extraction Method: Principal Component Analysis.

Figure B-1. A Scree Plot that shows the drastic decrease in the size of the Eigenvalues and helps determine the number of factors that accounts for most of the variation in the data

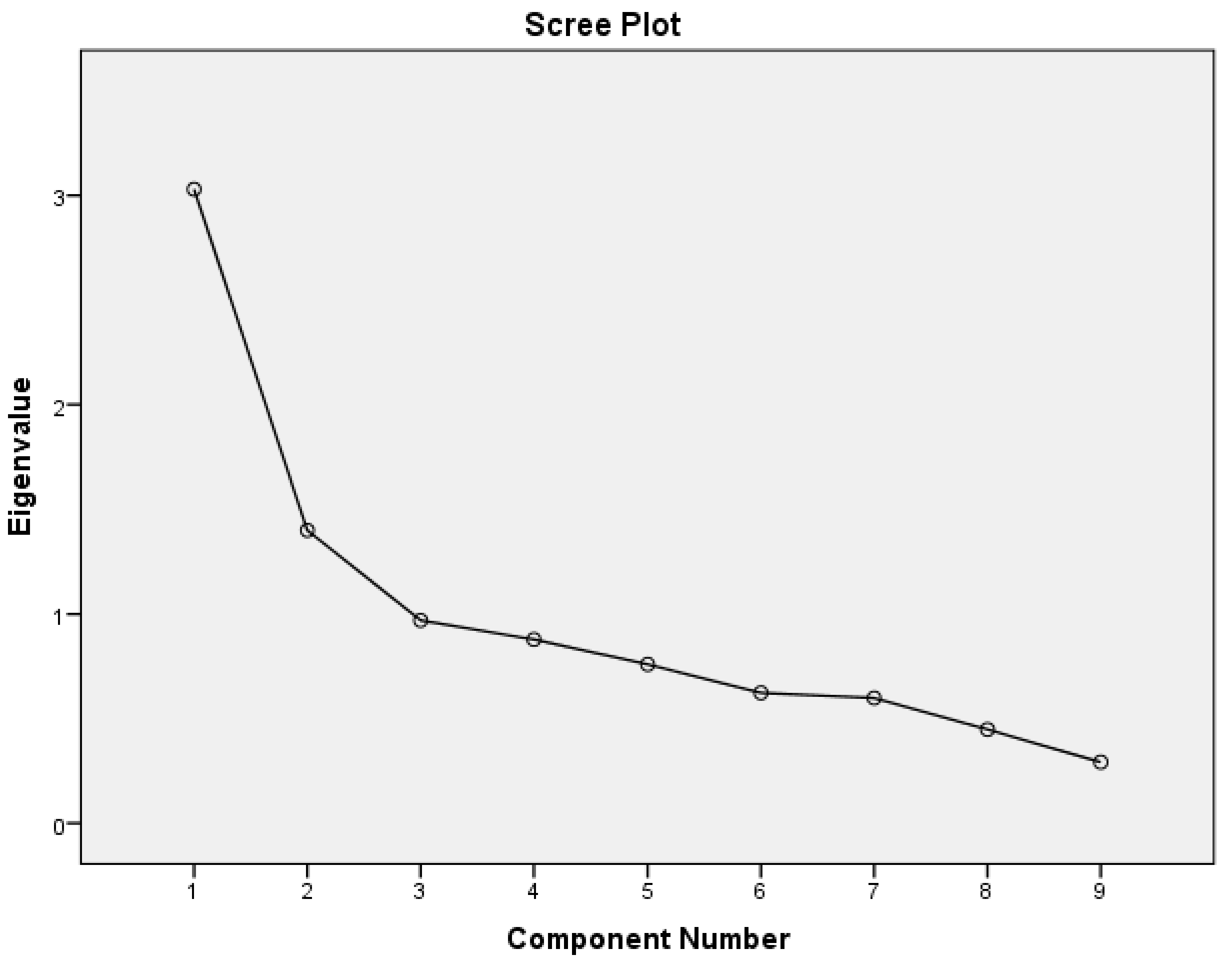

Table B-5. Initial Factor Matrix[a]

Variable	Component	
	1	2
Donning	.505	.150
Discomfort	.548	.505
Vision	.510	.454
Hearing	.694	-.454
Speech	.777	-.386
SA	.717	-.360
Thinking	.400	.376
Decisions	.483	-.200
Doffing	.470	.500

Extraction Method: Principal Component Analysis.

2 components extracted.

SA: Situational awareness

Table B-6. Rotated Component Matrix[a]

Variable	Component	
	1	2
Donning	.309	.427
Discomfort	.127	.734
Vision	.127	.671
Hearing	.827	.063
Speech	.851	.168
SA	.788	.152
Thinking	.087	.542
Decisions	.504	.136
Doffing	.068	.683

Extraction Method: Principal Component Analysis.

Rotation Method: Varimax with Kaiser Normalization.[a]

Rotation converged in 3 iterations.

SA: Situational awareness

This table shows the convergent and discriminant validity:

Table B-7. Component Transformation Matrix

Component	1	2
1	.793	.610
2	-.610	.793

Extraction Method: Principal Component Analysis.
Rotation Method: Varimax with Kaiser Normalization.
